# Integrated Analysis Reveals *ENDOU* as a Biomarker in Head and Neck Squamous Cell Carcinoma Progression

**DOI:** 10.3389/fonc.2020.522332

**Published:** 2021-02-05

**Authors:** Chengzhi Xu, Yunbin Zhang, Yupeng Shen, Yong Shi, Ming Zhang, Liang Zhou

**Affiliations:** ^1^ Department of Otolaryngology—Head and Neck Surgery, Eye Ear Nose and Throat Hospital, Fudan University, Shanghai, China; ^2^ Shanghai Institute of Biochemistry and Cell Biology, Chinese Academy of Sciences, University of Chinese Academy of Sciences, Shanghai, China; ^3^ Department of Respirology, Shanghai Public Health Clinical Center, Fudan University, Shanghai, China; ^4^ Department of Otolaryngology—Head and Neck Surgery, Bethune International Peace Hospital, Shijiazhuang, China

**Keywords:** head and neck squamous cell carcinoma, *ENDOU*, prognosis, tumor suppressor, bioinformatics

## Abstract

**Background:**

Head and neck squamous cell carcinoma (HNSCC) is a leading cancer with high morbidity and mortality worldwide. The aim is to identify genes with clinical significance by integrated bioinformatics analysis and investigate their function in HNSCC.

**Methods:**

We downloaded and analyzed two gene expression datasets of GSE6631 and GSE107591 to screen differentially expressed genes (DEGs) in HNSCC. Common DEGs were functionally analyzed by Gene ontology and KEGG pathway enrichment analysis. Protein-protein interaction (PPI) network was constructed with STRING database and Cytoscape. *ENDOU* was overexpressed in FaDu and Cal-27 cell lines, and cell proliferation and migration capability were evaluated with MTT, scratch and transwell assay. The prognostic performance of ENDOU and expression correlation with tumor infiltrates in HNSCC were validated with TCGA HNSCC datasets.

**Results:**

Ninety-eight genes shared common differential expression in both datasets, with core functions like extracellular matrix organization significantly enriched. 15 genes showed prognostic significance, and *COBL* and *ENDOU* serve as independent survival markers in HNSCC. In-vitro *ENDOU* overexpression inhibited FaDu and Cal-27 cells proliferation and migration, indicating its tumor-suppressing role in HNSCC progression. GSEA analysis indicated *ENDOU* down-stream pathways like DNA replication, mismatch repair, cell cycle and IL-17 signaling pathway. *ENDOU* showed relative lower expression in HNSCC, especially HPV-positive HNSCC samples. At last, *ENDOU showed* negative correlation with tumor purity and tumor infiltrating macrophages, especially M2 macrophages.

**Conclusion:**

This study identified *ENDOU* as a biomarker with prognostic significance in HNSCC progression.

## Introduction

HNSCC is a leading cancer with high morbidity and mortality worldwide (1, 2). It can be categorized by the area of the head or neck in which they begin, like oral cavity, pharynx, oropharynx, larynx, paranasal sinuses and nasal cavity and salivary glands. The most important pathogenic risk factors of HNSCC are tobacco and alcohol consumption and at least 75% of head and neck cancers are attributed to them (3). Molecular pathogenesis is still a comprehensive but largely not understood problem in HNSCC. Due to the phenotypic heterogeneity of each individual patient, it is hard to match suitable and effective treatments. Over the past decades, the development of high-throughput sequencing, increasing researchers have focused on the biological genetic functions of genes involved in the tumor-genesis of HNSCC (4–12).They have illustrated a lot of large-scale gene-expression profiles of HNSCC samples. The first successful therapeutic strategy for HNSCC was to inhibit the epidermal growth factor receptor (EGFR). In-depth understanding of the molecular carcinogenesis of HNSCC could help to develop novel and accurate treatment strategies for individual HNSCC patient.

The Cancer Genome Atlas (TCGA) network group of NIH generated comprehensive genomic characterization of HNSCC in 2015 (5). It profiled 279 HNSCC samples and provided a large-scale landscape of genetic and epigenetic characterizations of HNSCC, pointing the pivotal roles of *PIK3CA*, *TRAF3*, *E2F1*, *TP53*, *NOTCH* and other regulators in Wnt signaling pathways in tumor-genesis of HNSCC. Before that, Chen and his colleagues also compared 22-paired samples of HNSCC and normal tissues in 2004 (13). They found critically altered genes in the pathogenic processes of HNSCC through combinatorial analysis of microarray data. However, HNSCC is a cancer with complicated pathogenesis as well as diverse tissue origins. At present, most researches of HNSCC focus on star pathways or molecules, like PI3K signaling pathway or *TP53* protein. Therefore, it’s necessary and urgent to dig out more potential targets for HNSCC treatment.

In this study, we downloaded two published and well-generated profiling datasets about HNSCC tumor and normal tissues from the Gene Expression Omnibus (GEO). Then novel potential prognostic markers were dig out to find more avenues to effective clinical treatments of HNSCC *via* multiple bioinformatics analysis, including biological process functional annotation and pathway enrichment analysis, protein-protein interaction network analysis as well as gene expression profiling interactive survival analysis. Gene *ENDOU* showed differential expression, correlation with tumor infiltrates and prognostic significance, and were functionally investigated.

## Materials and Methods

### Data Collection

The expression profiles of RNAs were screened from the National Center of Biotechnology Information Gene Expression Omnibus (http://www.ncbi.nlm.nih.gov/gds/). The GSE6631 dataset is composed of 22 paired HNSCC tumor and normal samples. The platform is GPL8300 [HG_U95Av2] Affymetrix Human Genome U95 Version 2 Array. The GSE107591 dataset contains 23 normal and 24 tumor tissues. The platform is GPL6244 [HuGene-1_0-st] Affymetrix Human Gene 1.0 ST Array.

### Identification of Differentially Expressed Genes (DEGs)

We utilized quartile normalization algorithm to subtract and correct background of these downloaded datasets firstly (14). Then we filtered probes without corresponding gene symbols and calculated the average value of gene symbols with multiple probes. We further used *R* software *Limma* package to screen DEGs by filtering *p.adj* value of student’s t-test and fold change (FC) (15). Finally, with a threshold of p.adj-value <0.05 and absolute value of FC >2 (16, 17), volcano plot was performed by using R software ggplot2 package to identify the DEGs with statistical significance between two groups. Hierarchical clustering and combined analyses were performed for the DEGs.

### Functional Analysis of DEGs in HNSCC Pathogenesis

GO enrichment analyses of differentially expressed mRNAs were implemented with annotation, visualization and integrated discovery (DAVID) (http://david.abcc.ncifcrf.gov/). GO terms (including Molecular Function, Biological Processes and Cellular Components) with p-value less than 0.05 were considered significantly enriched by DEGs. KEGG is a database resource for understanding high-level functions and effects of the biological systems (http://www.genome.jp/kegg/). DAVID was also used to test the significantly statistical enrichment of DEGs in KEGG pathways. Cytoscape (version 3.40) was used to visualize the relationships between biological processes terms and DEGs.

### Protein-Protein Interaction (PPI) Network Analysis

Protein-protein interaction analysis was further conducted in STRING database (https://string-db.org/). The higher or larger score protein-protein interaction pairs were selected to construct PPI networks. Then, the regulatory relationships between genes were visualized *via* Cytoscape (version 3.4.0) (18). The sub-networks were extracted from the whole PPI network by using MCODE app.

### Survival Analysis and Tumor Filtrating Immune Cells Analysis

To conduct survival analysis, clinical data and RNA expression data from TCGA dataset were downloaded. Univariate and Multivariate Cox analysis was conducted with SPSS, and forest plot was conducted with R. Then Kaplan-Meier survival plot and LogRank analysis was done. The correlation of *ENDOU* expression with tumor infiltrating cells were conducted with TIMER (https://cistrome.shinyapps.io/timer/) (19). For macrophage markers, *CCL2*, *CD68*, and *IL10* were used for tumor associated macrophages (TAM), *NOS2*, *IRF5*, and *PTGS2* for M1 macrophages and *CD163*, *VSIG4*, and *MS4A4A* for M2 macrophages, referring to previous study (20).

### Cells Culture

The FaDu and Cal-27 human carcinoma cell lines were propagated and carefully maintained in our laboratory. FaDu and Cal-27 cells were cultured in Dulbecco’s modified Eagle’s medium supplemented with 10% fetal bovine serum at 37°C in a humid atmosphere containing 5% CO2.

### Lentivirus Mediated *ENDOU* Overexpression

CDS of *ENDOU* (NM_001172440) was inserted into a recombinant lentiviral expression vector (pGSIL) containing green fluorescent proteins (GFP) tag. To generate lentiviral particles, the recombinant expression plasmid was co-transfected with a packaging plasmid system (psPAX2 and pMD2G) into FaDu and Cal-27 cells, and viral particles were collected after 48 h. FaDu and Cal-27 cells were infected with *ENDOU* lentiviral vector or with a negative control (NC) for 24 h. The infection efficiency was preliminarily assessed in each experiment under a fluorescence microscope and then measured by sorting GFP-positive cells by flow cytometry (Beckman Coulter, USA). The stably infected cells were expanded and harvested for further experiments. The overexpression of *ENDOU* was examined with western blotting, using monoclonal antibody (Abcam, Cambridge, MA, USA). GAPDH was used as the house-keeping gene.

### MTT Assay

The cell proliferation activity of FaDu and Cal-27 cells was examined with MTT Cell Proliferation and Cytotoxicity Assay Kit (Dojindo Laboratories, Tokyo, Japan). The cells were incubated for 24, 48, 72, and 96 h. After incubation, the MTT solution was removed and replaced with dimethyl sulfoxide (DMSO; 150 μl, 4%; Sigma). A microplate reader (Bio-Tek, Instruments, Neufahrn, Germany) was used to measure the absorbance at 570 nm.

### Transwell Assay

Cells in logarithmic growth phase were seeded at the upper transwell chamber insert (Corning, USA) at a density of 2 × 104 cells per well. The chamber was placed in a 24- well plate in which the upper chamber contained serum‐free cell culture medium and the lower chamber contained 10% FBS complete medium. The culture was continued for 24 h. The medium was discarded, and stained with a crystal violet solution to observe the number of migrated cells.

### Scratch Wound Healing Assay

FaDu and Cal-27 cells were grown in complete growth medium until 90% confluence after transfection. A 3 mm wound was introduced across the diameter of each plate. Cell migration was observed by microscopy at 24 h.

### Statistical Analysis

Student’s t-test or ANOVA were performed to analyze the differential expression. Kaplan-Meier analysis was used to estimate overall survival rate or time; the differences between the survival curves were analyzed using the log-rank test. In all analyses, P<0.05 was considered to indicate a statistically significant result. Continuous data are presented as the mean ± standard deviation.

## Results

### DEGs Identification in Two HNSCC Datasets

**Figure 1:**
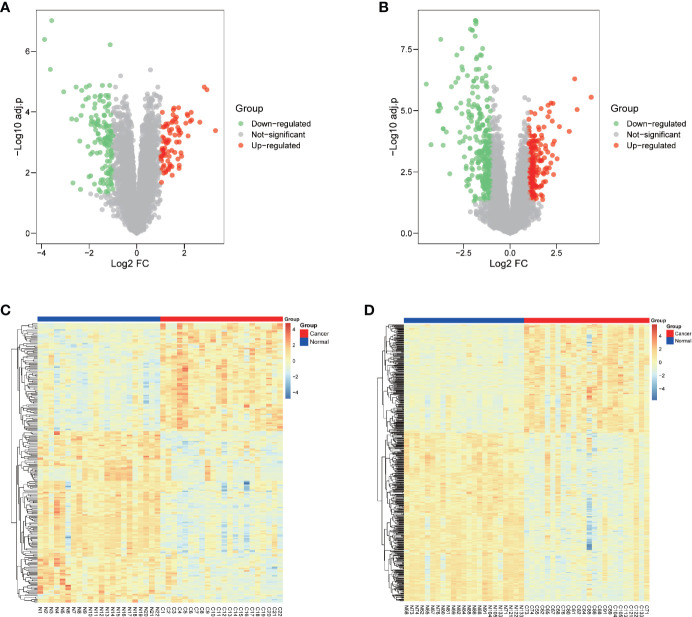
Differentially expressed genes (DEGs) of normal and Head and neck squamous cell carcinoma (HNSCC) tissue from GSE6631 and GSE107591. **(A)** Volcano plot showing significantly deregulated genes in GSE6631; **(B)** Volcano plot showing significantly deregulated genes in GSE107591; **(C)** Heatmap clustering of all DEGs in normal and HNSCC tissue from GSE6631; **(D)** Heatmap clustering of all DEGs in normal and HNSCC tissue from GSE107591; UP, genes with significant increased expression in HNSCC group were labeled in red. DW, genes with significant decreased expression in HNSCC group were labeled in green, genes with no different were in HNSCC group were labelled in grey.

Two GEO datasets of HNSCC, GSE6631 and GSE107591 were first normalized (**Figure S1**) and analyzed to acquire significantly differential expressed genes (DEGs), respectively. Both datasets contains more than twenty tumor and normal samples, and with the same microarray platform of Affymetrix gene expression microarray. The GSE107591 dataset contains 23 normal and 24 tumor tissues. As GSE6631 comprises 22 paired HNSCC tumor and normal samples, we conducted paired comparison. With a threshold of p.adj-value <0.05 and absolute value of FC >2, the expression of DEGs in HNSCC tumor and normal tissues were shown in **Figure 1**. We found 91 genes significantly up-regulated and 121 genes down-regulated in GSE6631 dataset (**Figures 1A, C**). For GSE107591, we found 194 up-regulated and 278 down-regulated genes in HNSCC tumor samples compared with normal tissues (**Figures 1B, D**).

### Functional Analysis of 98 Common DEGs Enriched Hub Pathways in HNSCC

**Figure 2:**
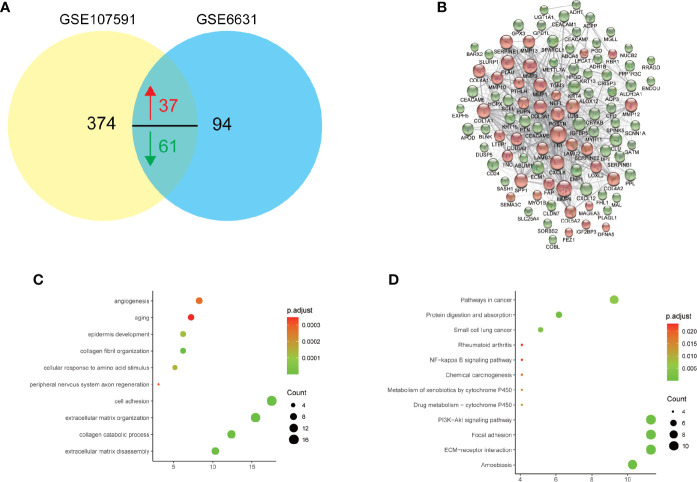
Gene Ontology (GO) and KEGG pathway analysis of 98 commonly deregulated genes in GSE6631 and GSE107591. P value, count and ratio of top 10 enriched functional items were shown. **(A)** Venn plot of 98 commonly deregulated genes in GSE6631 and GSE107591; **(B)** Protein-protein interaction (PPI) network of 98 commonly deregulated genes; **(C)** Top 10 enriched Gene Ontology biological processes, including extracellular matrix disassembly were significantly identified; **(D)** Top 10 enriched KEGG pathways including ECM-receptor interaction were shown.

To further figure out the roles of these DEGs in HNSCC, we searched the common DEGs both altered in these two datasets. A total of 98 DEGs (37 up-regulated and 61 down-regulated DEGs) showed dysregulation in both datasets (**Figure 2A**). To further detailed unravel the function of these common DEGs in HNSCC, we constructed protein-protein interaction (PPI) network *via* STRING database (https://string-db.org/). The genes with larger scores were selected to construct PPI networks (**Figure 2B**), and their function was uploaded to DAVID database for KEGG pathway and GO enrichment analysis. As shown in **Figure 2C**, biological processes with the largest number of common DEGs and smallest p-value includes extracellular matrix disassemble, collagen catabolic process, extracellular matrix organization, cell adhesion, aging and angiogenesis. As for the KEGG enrichment analysis in **Figure 2C**, not surprisingly, items like Pathways in cancer, ECM-receptor interaction, Focal adhesion and PI3K-Akt signaling pathways are significantly enriched and showed the largest number of common DEGs. The PI3K-Akt signaling was inappropriately activated in many cancers, including head and neck cancer (21). Somatic mutations of *PIK3CA* had been also described before and are found in about 15% of HNSCC (22–24). These findings might help us find more new therapy targets for HNSCC.

### Clinical Significance of 15 Genes Were Analyzed With TCGA HNSCC Datasets

**Figure 3:**
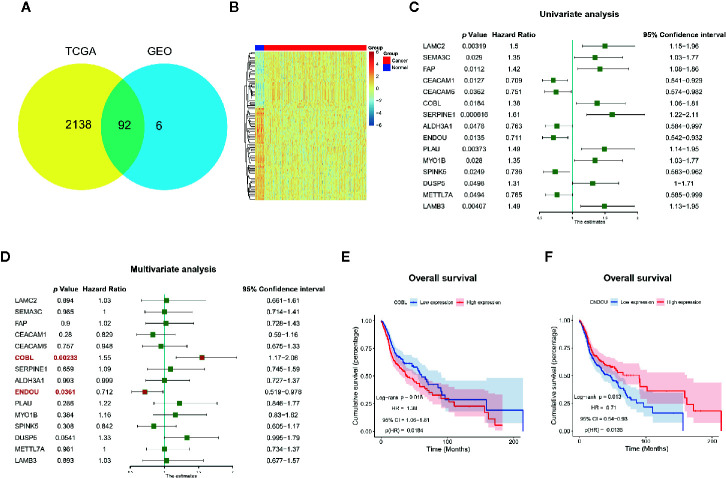
Prognostic significance of genes in The Cancer Genome Atlas (TCGA) HNSCC dataset. **(A)** Ninety-two genes showed consistent dysregulation in TCGA dataset, and were shown with Venn plot; **(B)** Heatmap of the dysregulated 92 genes in TCGA HNSCC cancer and normal samples; **(C)** Univariate analysis identified 15 genes with prognostic significance in TCGA HNSCC datasets; **(D)** Multivariate analysis further identified COBL and ENDOU as independent prognostic markers in HNSCC; **(E, F)** Kaplan-Meier overall survival plot of COBL **(E)** and ENDOU **(F)** in HNSCC.

To further explore the clinical significance of DEGs in HNSCC, we first validated the expression of 98 common DEGs with TCGA HNSCC dataset. A total of 2230 DEGs were acquired in HNSCC datasets and 92 genes (in 98 common DEGs, **Table S1**) showed significant dysregulation in all datasets (**Figures 3A, B**). Then the prognostic significance of the 92 DEGs were analyzed with univariate analysis. As shown in **Figure 3C**, 15 genes showed promising performance with HNSCC overall survival. Nine genes of *LAMC2* (HR=1.5, P=0.00319), *SEMA3C* (HR=1.35, P=0.029), *FAP* (HR=1.42, P=0.0112), *COBL* (HR=1.38, P=0.0184), *SERPINE1* (HR=1.61, P=0.000616),*PLAU* (HR=1.49, P=0.00373), *MYO1B* (HR=1.35, P=0.028), *DUSP5* (HR=1.31, P=0.0498), *LAMB3* (HR=1.49, P=0.00407) were hazardous, and 6 genes of *CEACAM1* (HR=0.709, P=0.00049), *CEACAM6* (HR=0.751, P=0.0362)*, ALDH3A1*(HR=0.763, P=0.0478), *ENDOU* (HR=0.711, P=0.0135)*, SPINK5* (HR=0.736, P=0.0249) and *METTL7A* (HR=0.765, P=0.0494) were potential protective markers. At last, the multivariate analysis was conducted and only *COBL* and *ENDOU* showed significance (**Figure 3D**), indicating *COBL* (HR=1.55, P=0.00233) and *ENDOU* (HR=0.712, P=0.0361) as independent prognostic overall markers in HNSCC, and the Kaplan-Meier overall survival plot of *COBL* and *ENDOU* was shown in **Figures 3E, F**, and other 13 genes were shown in **Figure S2**.

Cordon-Bleu WH2 Repeat Protein (*COBL*) contains WH2 domains (WASP, Wiskott-Aldrich syndrome protein, homology domain-2) and has been reported to play an important role in the reorganization of the actin cytoskeleton (25–27). In acute lymphoblastic leukemia, *COBL* is a hotspot for *IKZF1* deletions (28), and we will study the function of *COBL* in HNSCC in the future. *ENDOU*, also known as *PP11* (Placental Protein 11), encodes uridylate-specific endoribonuclease expressed in the placenta. *ENDOU was reported to* displays RNA binding capability and cleaves single stranded RNA in a Mn (2+)-dependent manner at uridylates (29). Studies of *ENDOU* in cancer dates back to 1980s (30, 31). *ENDOU* was annotated with the function of in proteolysis (Gene Ontology term GO:0006508), and members like matrix metalloproteinases (MMPs) play an important function in cancer progression, by depredating extracellular matrix. Considering the significant enrichment of functional items of extracellular matrix in **Figures 2C, D**, we chose *ENDOU* for the following study.

At last, the clinical association between ENDOU expression and clinicopathological variables in TCGA HNSCC patients was analyzed. As shown in **Table 1**, ENDOU expression showed significant correlation with survival status (P=0.03), gender (P=0.011), alcohol history (P=0.017), clinical N stage (P=0.001), lymphovascular invasion status (P=0.001), neoplasm histologic grade (P<0.001) and pathologic N stage (P<0.001). In sum, *ENDOU* may serve as an independent prognostic marker in HNSCC.

**Table 1:** Clinical association between ENDOU expression and clinicopathological variables in HNSCC patients.

Characteristics	n	No. of Patients (%)	ENDOU		
			Low	High	*p*
**Status**	500				0.03
Alive		282(56.4)	129	163	
Death		218(43.6)	121	97	
**Gender**	500				0.011
Female		133(26.6)	54	79	
Male		367(73.4)	196	171	
**Age**	499				0.616
<60		220(44.1)	107	113	
≥60		279(55.9)	142	137	
**Alcohol_history**	489				0.017
No		157(32.1)	66	91	
Yes		332(67.9)	178	154	
**Clinical_N**	478				0.001
N0		239(50)	101	138	
N1-3		239(50)	139	100	
**Clinical_T**	485				0.731
T1-2		176(36.3)	86	90	
T3-4		309(63.7)	156	153	
**Clinical_stage**	486				0.096
I-II		114(23.5)	49	65	
III-IV		372(76.5)	193	179	
**Lymph_node_count**	407				0.52
Low		203(49.9)	102	101	
High		204(50.1)	96	108	
**Lymphovascular_invasion_present**	339				0.001
No		219(64.6)	95	124	
Yes		120(35.4)	74	46	
**Neoplasm_histologic_grade**	481				0
G1-2		360(74.8)	151	209	
G3-4		121(25.2)	88	33	
**Recurrence**	442				0.39
No		317(71.7)	153	164	
Yes		125(28.3)	66	59	
**Pathologic_N**	407				0
N0		171(42.0)	63	108	
N1-3		236(58.0)	132	104	
**Pathologic_T**	444				0.799
T1-2		177(39.9)	84	93	
T3-4		267(60.1)	130	137	
**Pathologic_stage**	435				0.133
I-II		98(22.5)	41	57	
III-IV		337(77.5)	170	167	
**Perineural_invasion**	351				0.311
No		186(53.0)	88	98	
Yes		165(47.0)	87	78	
**Smoker**	490				0.914
No		111(22.7)	56	55	
Yes		379(77.3)	189	190	

### *ENDOU* Inhibits HNSCC Cancer Cell Proliferation and Migration *In Vitro*

**Figure 4:**
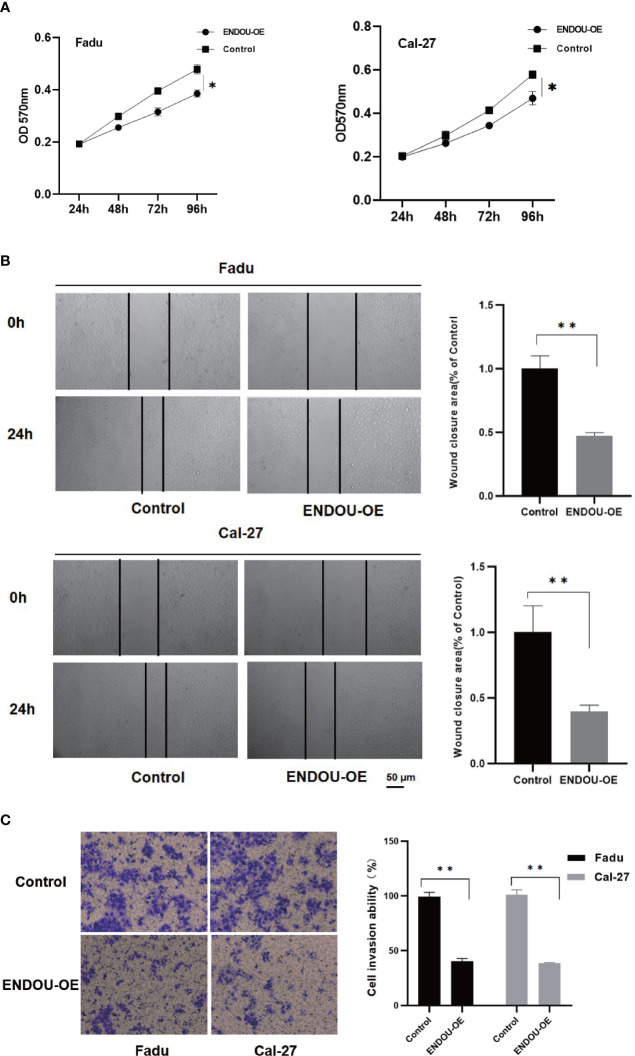
ENDOU overexpression inhibits Fadu and Cal-27 cells proliferation and migration *in vitro*. **(A)** MTT assay results indicated *ENDOU* overexpression (OE) inhibited Fadu and Cal-27 cells proliferation. Each assay was replicated three times. *P < 0.05. **(B)** Scratch healing assay showed decreased migration capability in *ENDOU* overexpressing Fadu and Cal-27 cells. Each assay was replicated three times. *P < 0.05. **(C)** Transwell assay showed that *ENDOU* overexpression (OE) attenuated the invasion capability of Fadu and Cal-27 cells. Each assay was replicated three times. *P < 0.05, **P < 0.01.

As *ENDOU* shows consistent lower expression and hazardous prognostic significance in HNSCC, to explore the function of *ENDOU* in HNSCC, we conducted in-vitro over-expression (OE) studies in two cell lines of Fadu and Cal-27. The proliferation and migration capability was examined. As shown in **Figure 4A**, the proliferation capability of Fadu and Cal-27cells in *ENDOU* overexpression group was significantly decreased. Then wound healing assay and transwell assay was applied to study the effect of *ENDOU* overexpression on cell migration and invasion. In **Figures 4B, C**, the migration distance of *ENDOU* overexpression group was significantly larger than vector groups, and cells numbers was significantly decreased, implying that *ENDOU* may serve as a tumor suppressor in HNSCC.

**Figure 5:**
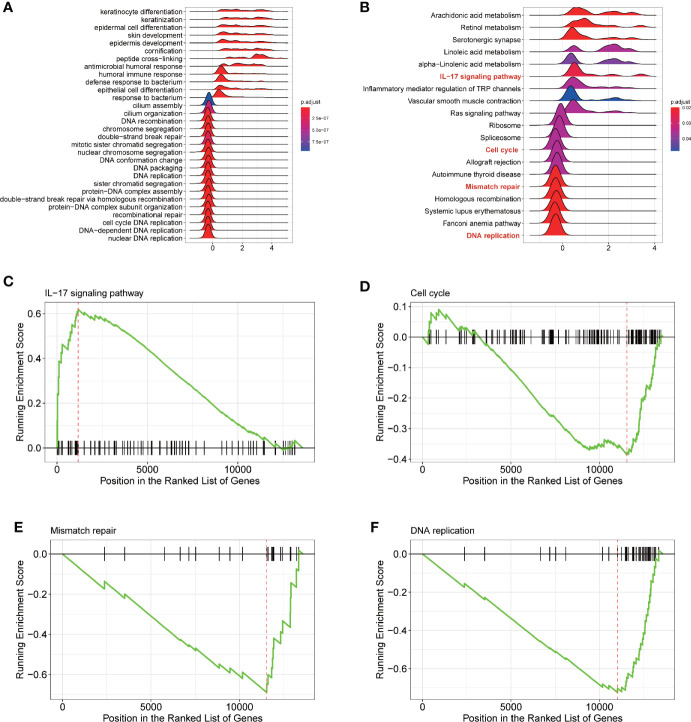
Gene Set Enrichment Analysis (GSEA) analysis reveals DNA replication, Mismatch repair, Cell cycle and IL-17 signaling pathway as potential ENDOU functional mechanism. **(A)** GSEA analysis of Gene ontology enrichment items of ENDOU; **(B)** GSEA analysis of KEGG pathway enrichment items of ENDOU; **(C)** GSEA Enrichment plot of IL-17 signaling pathway in head and neck squamous cell carcinoma (HNSCC); **(D)** GSEA Enrichment plot of Cell cycle in HNSCC; **(E)** GSEA Enrichment plot of Mismatch repair in HNSCC; **(F)** GSEA Enrichment plot of DNA replication in HNSCC.

To explore the potential mechanism of *ENDOU* as a tumor suppressor in HNSCC, we applied Gene Set Enrichment Analysis (GSEA) analysis to enrich *ENDOU* related KEGG pathways and biological processes. As shown in **Figure 5**, DNA replication, mismatch repair, cell cycle and IL-17 showed significant enrichment, which supported the cellular phenotype of *ENDOU* in HNSCC.

### *ENDOU* Expression Showed Significant Correlation With Macrophages in HNSCC

**Figure 6:**
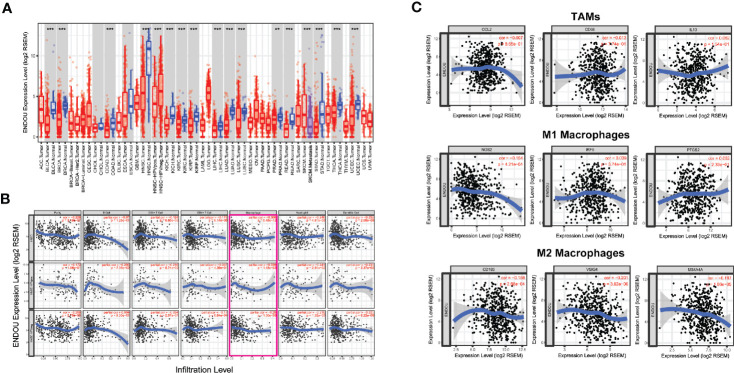
*ENDOU* showed significant down-regulation in head and neck squamous cell carcinoma (HNSCC) development and correlates with tumor infiltrating immune cells. **(A)** *ENDOU* was significantly downregulated in HNSCC samples, compared with normal tissue. *ENDOU* showed significant lower expression in HPV-positive HNSCC samples. Decreased expression was also observed in bladder cancer (BLCA), breast cancer (BRCA), liver cancer (LIHC) and other cancer samples. **P < 0.01; ***P < 0.001; **(B)** The correlation of *ENDOU* expression with tumor infiltrates were analyzed, and *ENDOU* expression showed significant negative correlation with tumor purity, macrophages, neutrophils and dendritic cells; **(C)** *ENDOU* expression showed negative correlation with M2 macrophages markers, including *CD163*, *VSIG4*, and *MS4A4A*.

In addition to HNSCC, we analyzed the expression of *ENDOU* in other cancer types, as shown in **Figure 6A**, decreased expression was also observed in bladder cancer (BLCA), breast cancer (BRCA), liver cancer (LIHC) and other cancer samples, indicating a universal role of *ENDOU in cancer. For HNSCC*, Human papillomavirus (HPV) positive HNSCC has a far better prognosis than HPV negative HNSCC, and HPV infection has been linked with intratumoral immune cell infiltrates (e.g. IL-17+CD8+T lymphocytes) in HNSCC (32, 33). Then we compared the expression of *ENDOU* in HPV-negative and HPV-positive HNSCC samples, and *ENDOU* showed significant lower expression in HPV-positive HNSCC samples (**Figure 6A**). At last, we analyzed the expression of *ENDOU* with tumor immune infiltrates, in HPV-negative and HPV-positive HNSCC samples. As shown in **Figure 6B**, *ENDOU* showed significant negative correlation with neutrophils, dendritic cells and especially macrophages. As the correlation was most significant in tumor infiltrating macrophages, correlation of TAMs, M1 and M2 macrophages markers with *ENDOU* expression were analyzed. As shown in **Figure 6C**, the markers of M2 macrophages (*CD163*, *VSIG4*, and *MS4A4A*) all showed significant negative correlation coefficient with *ENDOU*, implicating potential role of *ENDOU* in tumor infiltrating M2 macrophages.

## Discussion

At present, head and neck cancer is the sixth most common cancer with a poor prognosis and high mortality over the world accounting for approximately 4% of all cancers in the United States (34). HNSCC is a kind of highly heterogeneous malignancy and appropriate clinical treatments remain a major challenge for HNSCC because of heterogeneity. Hence, personalized care for HNSCC patients requires a better understanding of the molecular mechanism of HNSCC. Though some biomarkers in head and neck squamous cell carcinoma progression have been reported yet (35, 36), accepted biomarkers for HNSCC prognosis in the clinic is still raw. Effective prognostic care for HNSCC patients requires a better understanding of the molecular mechanism. With the recent development of next generation sequencing and other “omics” profiling methods, we began to focus on genomic analysis of HNSCC to illustrate the detailed or new causes of HNSCC pathogenesis and to try to develop new markers for treatments of this cancer.

In the present study, gene expression data of HNSCC were downloaded from GEO, which revealed significant differences between survival status and clinical treatments of HNSCC patients. Hence, we reanalyzed two published microarray datasets, GSE6631 and GSE107591 in this study. Finally, we found 98 common DEGs between 45 normal and 46 HNSCC tumor samples. Gene functional annotation and pathway enrichment analysis of these DEGs showed that extracellular matrix disassemble collagen catabolic process, extracellular matrix organization, cell adhesion, aging and angiogenesis were enriched in HNSCC. These results revealed that our bioinformatics analysis may help a better understanding of the regulation of these genes in HNSCC. There are also many researchers focusing on extracellular matrix organization play an important role in HNSCC (37, 38). They pointed out that extracellular matrix organization was one of the most frequently altered pathways in HNSCC, consistent with our results.

By integrating the clinical information in TCGA, we screened 9 hazardous and 6 protective prognostic markers. Of the 15 genes, *LAMC2* (39–41), *SERPINE1* (41–43), *PLAU* (44), *MYO1B* (45), *LAMB3* (46), *CEACAM1* (41, 47), *CEACAM6* (48, 49), *ALDH3A1* (50) and *SPINK5* (51) has been reported as HNSCC prognostic markers, and in this study, we first uncovered the clinical significance of *SEMA3C*, *FAP*, *COBL*, *DUSP5*, *ENDOU* and *METTL7A* with HNSCC survival. Semaphorin 3C (*SEMA3C*) has been reported to play a pivotal role in tumor microenvironment driven neuroblastoma metastasis and progression (52). Fibroblast Activation Protein Alpha (*FAP*) was proven to serve as a marker in metastatic prostate cancer (53), pancreatic cancer (54) and ovarian cancer (55). Cordon-Bleu WH2 Repeat Protein (*COBL*) has been reported to play an important role in the reorganization of the actin cytoskeleton (25–27), and as a hotspot for *IKZF1* deletions (28) in acute lymphoblastic leukemia. Dual Specificity Phosphatase 5 (*DUSP5*) is tumor suppressor in ovarian cancer (56). Methyltransferase Like 7A (*METTL7A*) promoted cell viability and reduced apoptosis following methotrexate in choriocarcinoma (57). Further functional and mechanism studies of *SEMA3C*, *FAP*, *COBL*, *DUSP5*, *ENDOU*, and *METTL7A* may provide more detailed information to reveal their potential role as therapeutic targets in HNSCC.

Then, *ENDOU* not only showed significant lower expression in HNSCC cancer samples (compared with normal samples), but also decreased level in higher stage cancer samples, implicating its tumor suppressing function in HNSCC occurrence and progression. *ENDOU*, also known as *PP11* (Placental Protein 11), encodes uridylate-specific endoribonuclease expressed in the placenta. *ENDOU was reported to* displays RNA binding capability and cleaves single stranded RNA in a Mn (2+)-dependent manner at uridylates (29). Studies of *ENDOU* in cancer dates back to 1980s (30, 31), and this is the first time investigating the role of *ENDOU* in HNSCC. In this study, *ENDOU* was negatively correlated with tumor purity and then were functionally investigated in cancer cell lines. MTT, scratch healing and transwell assay demonstrated that *ENDOU* indeed inhibited the proliferation and migration capability, supporting it tumor suppressing role in HNSCC cancer cells. Meanwhile, there are several limits in this part. First, the effect of *ENDOU* overexpression can be validated in other HNSCC cell lines, and if possible, the impact of *ENDOU* silencing or mutation on HNSCC cell lines to support the conclusion. Secondly, an *in vivo* study of *ENDOU* would provide more solid evidence, as the function of genes *in vitro* and *in vivo* are not always consistent. Lastly, the underlying mechanism of *ENDOU* inhibiting FaDu cells proliferation and migration may be explored.

At last, tumor infiltrating immune cells in tumor microenvironment plays a pivotal role in HNSCC progression and prognosis prediction (58, 59). In this study, we found *ENDOU* negative correlates with tumor infiltrating neutrophils, dendritic cells and macrophages, especially M2 macrophages. Macrophages were reported to be involved in HNSCC metastasis (60–62), chemotherapy (63) and prognosis (64). Macrophages were polarized into M1 and M2 subtypes depending on their environment (65), and were reported to be functionally distinct in cancer (66). M2 polarized macrophages were found to be correlated with poor prognosis in nasopharyngeal carcinoma patients (67). HNSCC cancer cells were found to contribute to M2 polarization in several ways, like secreting microRNA-21 abundant exosomes (68) and lactic acid (69). Based on the founding that tumor suppressor gene *ENDOU* was negatively correlated with M2 markers of *CD163*, *VSIG4*, and *MS4A4A*, we speculated that *ENDOU* may also inhibit macrophages M2 polarization in tumor microenvironment.

In sum, our multiple bioinformatics analysis identified 98 genes with core functions like extracellular matrix organization significantly enriched. 15 genes showed prognostic significance, and *COBL* and *ENDOU* serve as independent survival markers in HNSCC. In-vitro *ENDOU* overexpression inhibited FaDu and Cal-27 cells proliferation and migration, indicating its tumor-suppressing role in HNSCC progression. GSEA analysis indicated *ENDOU* down-stream pathways like DNA replication, mismatch repair, cell cycle and IL-17 signaling pathway. *ENDOU* showed relative lower expression in HNSCC, especially HPV-positive HNSCC samples. At last, *ENDOU showed* negative correlation with tumor purity and tumor infiltrating macrophages, especially M2 macrophages. It was the first time that *ENDOU* was identified as a tumor suppressor, with prognostic significance and negative correlation with tumor infiltrating M2 macrophages. However, further biological and basic studies are needed to validate our findings.

## Data Availability Statement 

All datasets generated for this study are included in the article/**Supplementary Material**.

## Ethics Statement

The experiment was approved the Ethics Committee of Eye Nose and Throat Hospital, Fudan University.

## Author Contributions

CX, YZ, and LZ conceived and designed the study. YZ analyzed the data. YPS, YS, and MZ performed the experiments. YZ and LZ wrote the paper. CX and YPS reviewed and edited the manuscript. All authors contributed to the article and approved the submitted version.

## Funding

This study was supported by Natural Science Foundation ofShanghai (16ZR1419300), the National Natural Science Foundation (81972529) and Science and Technology Commission of Shanghai Municipality (9411961300).

## Conflict of Interest

The authors declare that the research was conducted in the absence of any commercial or financial relationships that could be construed as a potential conflict of interest.

## Supplementary Material

Supplementary Figure 1 Normalization and MDS plot of GSE6631 and GSE107591; **A, B**) Normalization boxplot of GSE6631 and GSE107591; **C, D**) MDS plot of GSE6631 and GSE107591.

Supplementary Figure 2 Kaplan-Meier survival plot of **(A)** PLAU; **(B)** ALDH3A1; **(C)** SERPINE1; **(D)** CEACAM6; **(E)** CEACAM61; **(F)** LAMB3; **(G)** METTL7A; **(H)** DUSP5; **(I)** SPINK5; **(J)** MYO1B; **(K)** FAP; **(L)** SEMA3C; **(M)** LAMC2 in HNSCC.
