# Supplementary figures and images for: Integrated Analysis Reveals ENDOU as a Biomarker in Head and Neck Squamous Cell Carcinoma Progression
Source: Front Oncol. 2021 Feb 5;10:522332. doi: 10.3389/fonc.2020.522332 (PMC7894080; doi:10.3389/fonc.2020.522332)

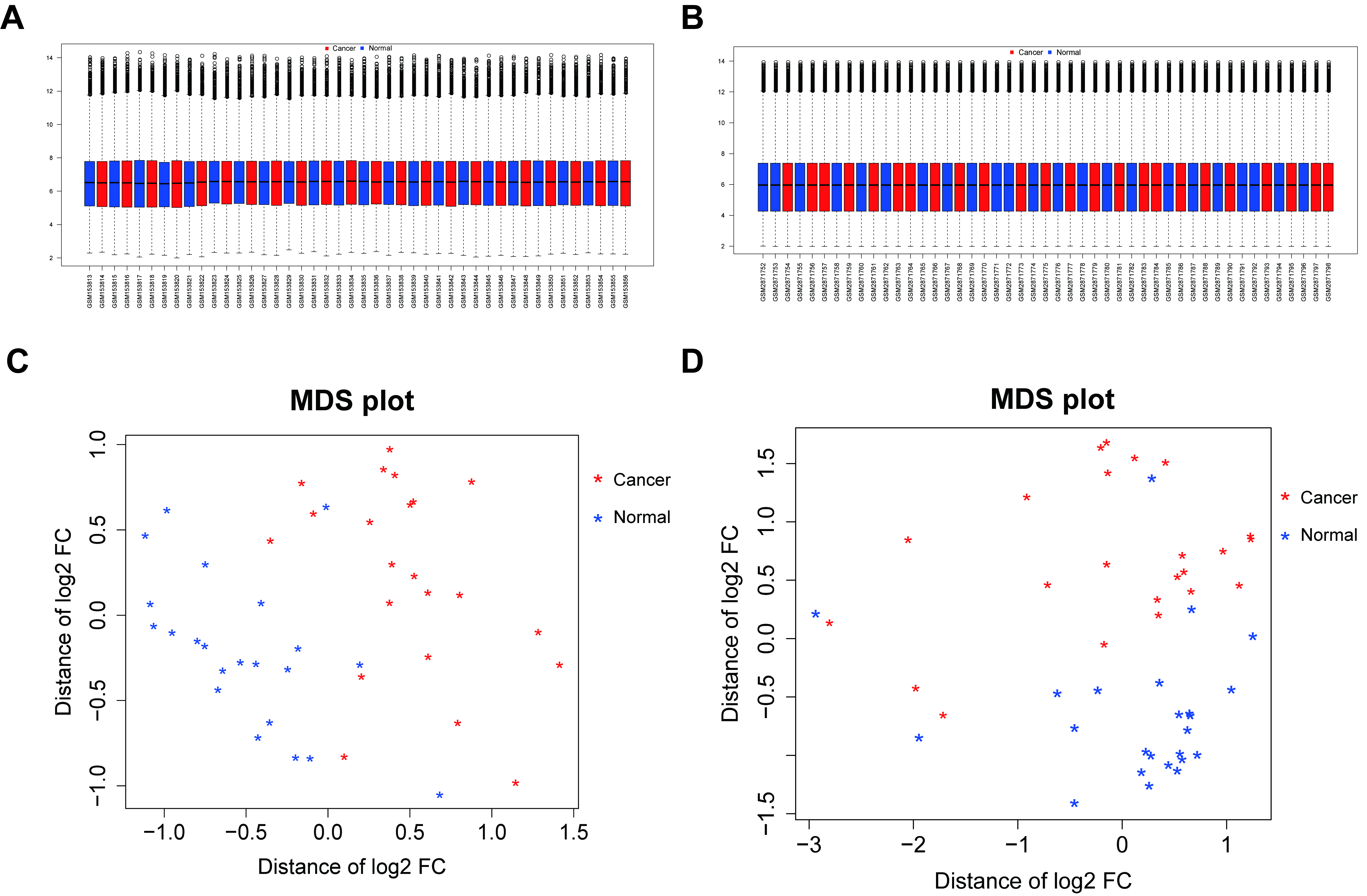

Supplement: Supplementary Figure 1 — Normalization and MDS plot of GSE6631 and GSE107591; A, B) Normalization boxplot of GSE6631 and GSE107591; C, D) MDS plot of GSE6631 and GSE107591. [file Image_1.tif]

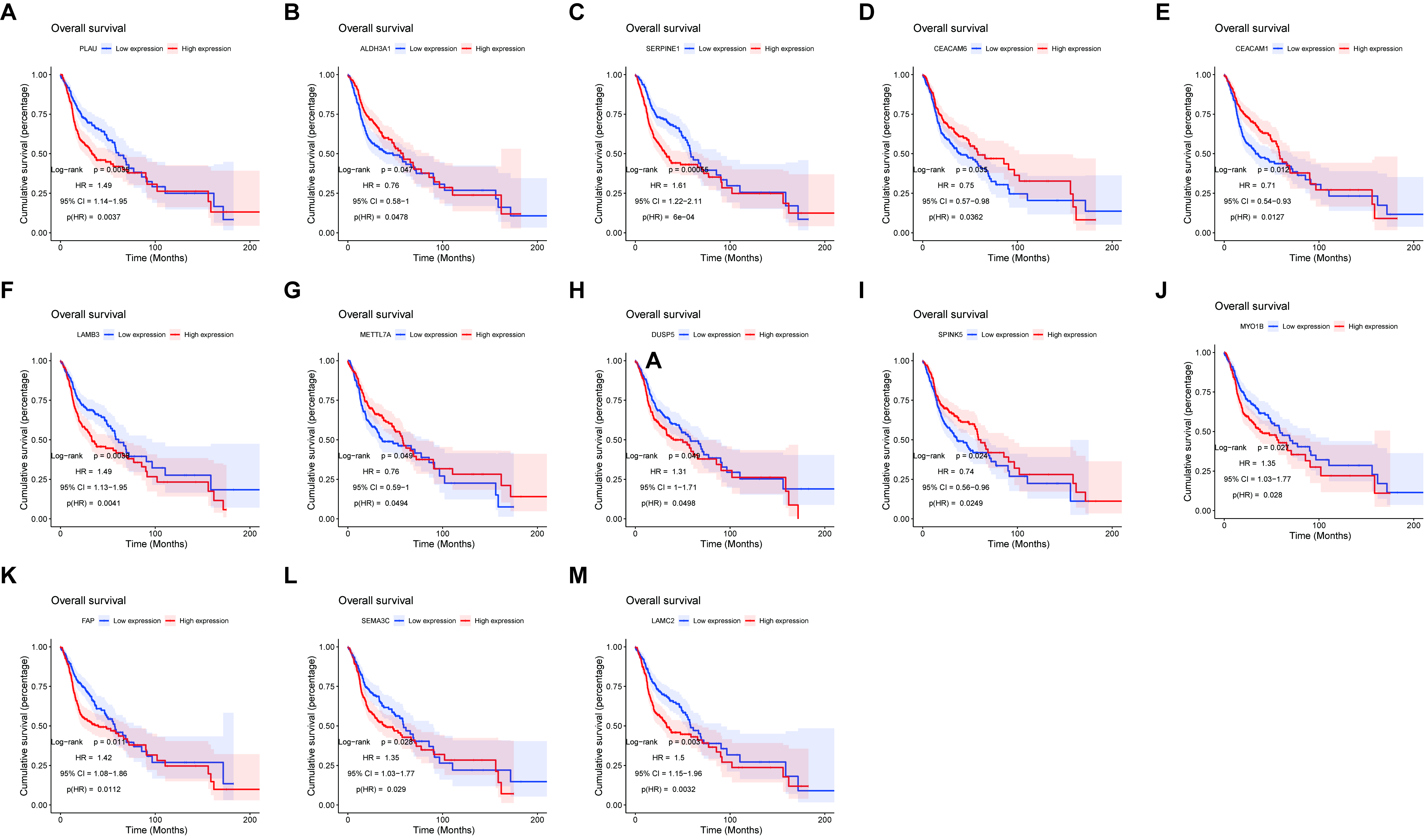

Supplement: Supplementary Figure 2 — Kaplan-Meier survival plot of (A) PLAU; (B) ALDH3A1; (C) SERPINE1; (D) CEACAM6; (E) CEACAM61; (F) LAMB3; (G) METTL7A; (H) DUSP5; (I) SPINK5; (J) MYO1B; (K) FAP; (L) SEMA3C; (M) LAMC2 in HNSCC. [file Image_2.tif]
